# Mental Health Recovery Using the Individual Recovery Outcomes Counter (I.ROC) in a Community Rehabilitation Team: A Service Evaluation

**DOI:** 10.1007/s40737-022-00315-2

**Published:** 2022-11-16

**Authors:** Angela L. Baufeldt, David L. Dawson

**Affiliations:** College of Social Sciences, University of Lincoln, Lincoln, UK

**Keywords:** Community rehabilitation team, Adult, Mental health, Recovery, Service evaluation

## Abstract

There are many definitions of recovery in mental health. Community Rehabilitation Teams (CRTs) aim to support the mental health recovery of people. The Individual Recovery Outcomes Counter (I.ROC) is a way to measure recovery. To determine if being supported by a CRT helps mental health recovery for people transitioning from an inpatient service to the community. Individual reliable and clinically meaningful change indices were calculated for a total of 31 people. Two I.ROC questionnaires were completed by 31 people. Of these 31 people, 14 people had three completed I.ROC questionnaires. Of the 31 people, 17 showed a positive reliable change and three people made a clinically meaningful change. Of the 14 people, one had a positive reliable change, two had a negative reliable change, and no-one had a clinically meaningful change. The I.ROC shows the CRT to successfully support recovery in people with mental health difficulties.

## Introduction

### Recovery in Mental Health

Mental health recovery can be considered from an individual level, i.e., personal recovery, but it can also be considered at a service level, for example a service employing a ‘recovery-oriented’ approach (Shields-Zeeman et al. 2020). This recovery-oriented approach at a service level tends to focus on clinical recovery i.e., symptom reduction, contrary to the principles of person-centred care (McKenna et al. 2016). This approach of focusing on symptom reduction is changing to more effective outcome measures of recovery in mental health services although, the lack of an agreed upon definition of mental health recovery hampers the adoption of more appropriate measures (Perkins and Slade 2012). The recovery concept in mental health, at an individual level, has largely been led by Anthony’s (1993) influential paper. In it, Anthony (1993) offered the following definition of recovery in mental health: “a deeply personal, unique process of changing one’s attitudes, values, feelings, goals, skills, and/or roles. It is a way of living a satisfying, hopeful, and contributing life even with limitations caused by illness” (p. 15). Despite the significant contribution of Anthony’s (1993) paper to the research generated in the field of mental health recovery, it largely neglects the social and environmental contributions to mental health difficulties. Karadzhov (2021) argues that mental health recovery is not entirely dependent on the individual changing their outlook, as Anthony’s definition of recovery would suggest, but that contextual factors such as homelessness, poverty, and other indicators of social exclusion are barriers to an individual’s recovery journey. NICE guidelines, in lieu of an agreed upon definition of mental health recovery, offer a recovery principle such that recovery is “the belief that it is possible for someone to regain a meaningful life, despite serious mental illness” (National Institute for Health and Care Excellence 2020 p. 44).

Since Anthony’s (1993) definition of recovery there have been many studies published exploring the concept of recovery in mental health (for a review see Llewellyn-Beardsley et al. 2019): in particular for adults with mental health difficulties (Dell et al. 2021). In this systematic review of 25 studies by Dell and colleagues (2021), they developed a model of recovery using reflexive thematic analysis. Recovery was defined as a process of transformation from a negative state of despair and powerlessness, to a positive state of psychological wellbeing. Although this definition suggests a close affiliation with Anthony’s (1993) definition, the authors go further and highlight the importance of socio-environmental factors, belonging, acceptance and insight, and autonomy and control (Dell et al. 2021). This study highlights the necessity of a holistic, person-centred, and individualised approach to mental health recovery. Recovery is therefore not only an intra-psychic process but requires social determinants of health, for example adequate housing, safety, food, income, and material resources incorporated into any recovery plan.

Similarly, rehabilitation is another concept that requires clarification. This service evaluation describes recovery as the way an individual learns to live with their mental health difficulties, while the support around the person is described as rehabilitation to facilitate recovery (Anthony 1993; Lloyd et al. 2008). Rehabilitation is a holistic and individually tailored approach that can take into account a person’s specific needs in order to facilitate the best possible conditions for a person to work towards their recovery goals.

### Recovery in Mental Health Services

Integrating a recovery approach into mental health services has been a goal of mental health policy in England since 2001 (Department of Health 2001; Perkins & Slade 2012). The absence of an agreed upon definition for recovery has consequently led to a lack of guidelines to direct this particular service provision. Service managers therefore, develop their service with consideration of the needs of their local population (Killaspy et al. 2005; National Institute for Health and Care Excellence 2016). The heterogeneity of rehabilitation service provision in England has been described in a national survey that found, of the 73 mental health trusts covering 93 boroughs who have mental health rehabilitation provision, 30% defined rehabilitation in terms of quality of life, 26% as maximising skills, and 25% in terms of a recovery model (Killaspy et al. 2005). This suggests that not all rehabilitation services are being defined in the same way. Furthermore, these rehabilitation services differed in their setting (residential, inpatient, respite, and community), and length of stay (long term, short term, or a mix of both). This heterogeneity in service provision for recovery in mental health is unsurprising as each service adopts different definitions of rehabilitation, and adapts to the resources they have in order to meet the demands of their local population.

### Community Rehabilitation Team

The NHS Long Term Plan (2019) outlines the goals of the NHS in England for the next 10 years and describes how funding will be used to achieve these goals. One of the goals is to improve community-based care for people who experience severe mental health difficulties (NHS 2019). This includes the development of integrated place-based community mental health teams that have improved co-ordination between primary care and health and social care. Recovery in mental health is an integral part of this plan and includes supporting people with employment, housing, and overcoming stigma (Mental Health Taskforce 2016). From this framework came the starting point of community rehabilitation teams (CRTs) in the local area and across the country (Killaspy et al. 2005). The purpose of CRTs is to provide intensive and individualised support for people transitioning from inpatient services to the community who may need additional support over and above community mental health teams (CMHTs); low volume, high needs (“Guidance for Commissioners of Rehabilitation Services for People with Complex Mental Health Needs: Volume Two: Practical Mental Health Commissioning” 2012).

CRTs need to adapt and be responsive to the needs of those they support. For example, within the local area, 48% of the population live in rural areas, with rural areas comprising 95% of the land (Rhodes 2018). Age-standardised suicide rates per 100,000 people between 2018 and 2020 were higher in the local area, than for England and Wales, 12.6 compared to 10.4, respectively (Nasir et al. 2021). This is consistent with the results of a systematic review which showed evidence of higher suicide rates in rural areas compared to urban areas (Casant and Helbich 2022). The review highlighted factors such as social isolation, access to lethal means, and reduced mental health services as being particular to a rural context. This CRT therefore, has an important role to play in supporting people to re-integrate into their communities especially given the unique challenges of a rural context.

### Measuring Recovery

Many measures of recovery have been developed over the years. A recent systematic review of mental health recovery measures by Penas and colleagues (2019) found 53 different measures available. Despite the large number of measures, only eight were deemed adequate for assessing mental health recovery in individuals (Penas et al. 2019). The eight identified measures by Penas and colleagues (2019) included published psychometric properties, quantitative data, included service users’ perspectives, limited to 50 items or less, and explicitly measured areas related to mental health recovery. Within this list, the Individual Recovery Outcomes Counter (I.ROC; Monger et al. 2013) was included alongside other measures widely used in the UK such as The Recovery Assessment Scale (RAS; Corrigan et al. 1999) and The Stages of Recovery Instrument (Shanks et al. 2013). A key highlight of this paper was the lack of consensus for what constitutes recovery in mental health and therefore the large number of measures (Penas et al. 2019).

The I.ROC was developed in Scotland in a sample of adults supported by community mental health teams (Monger et al. 2013). The impetus for the development of the I.ROC was the lack of available measures with evidence of measuring personal recovery that was suitable for routine use and validated in a UK population (Monger et al. 2013). During the development of the I.ROC, participants chose one of three measures; I.ROC, RAS, and the Behaviour and Symptom Identification Scale (BASIS-32; Eisen et al. 2007) they most preferred. Significantly more people chose the I.ROC as their preferred measure when compared to the RAS or the BASIS-32 and overall, 52% of participants chose the I.ROC as their favourite measure of recovery. Reasons for the preference included, the I.ROC being straightforward to complete and assisting participants to think about their recovery (Monger et al. 2013). The I.ROC has since undergone further psychometric testing (Dickens et al. 2017), including validation in different clinical populations such as those who experienced psychological trauma (Rudd et al. 2020), people with hearing difficulties (Roze et al. 2020), and has been translated into Dutch for adults with severe mental health illness (Beckers et al. 2022). The I.ROC therefore, has been shown to be a process measure of mental health recovery, which is suitable for regular use, preferred by individuals supported by mental health services, and developed in a UK population.

When analysing group data, significance testing is helpful to determine whether a change has occurred that is assumed to be the result of the manipulation of variables under investigation (Field 2016). This is a common method of analysing data and has been used in the investigation of recovery in mental health (van Aken et al. 2021). However, the use of significance testing in the field of psychology and, specifically the emphasis on the *p* value, has increasingly come under criticism (Hubbard et al. 2000; Hubbard & Lindsay 2008). Given the focus on assessing personal mental health recovery, individual reliable and clinically meaningful change indices have a much greater utility than group significance testing (Evans et al. 1998; Jacobson and Truax 1991). This type of analysis has the benefit of demonstrating whether observed changes are reliable, i.e., an individual has improved, and if so, whether they are clinically meaningful, i.e., an individual has recovered (Newnham et al. 2007). Where clinical and non-clinical distributions overlap, an individual’s change in a score may indicate improvement but not a sustained change. Therefore, the reliable change index (RCI) is calculated to assess whether or not a change is sustained and not due to random fluctuation. Clinically significant change (CSC) indicates when an individual’s score on a measure moves closer to the mean of a non-clinical population and away from the mean of the clinical population; in effect they have recovered. For an individual’s change to be clinically meaningful it must first be shown to be reliable.

The aim of this service evaluation is to determine if being supported by a CRT helps mental health recovery for people transitioning from an inpatient service to living in the community, as evidenced by improvements on the I.ROC measure. To reflect the different perspectives in the literature of individual versus service level perspective of recovery, the following objectives were developed to meet this aim.

1. To use individual reliable and clinically meaningful change indices from I.ROC data to determine mental health recovery in individuals supported by the CRT.
2. To evaluate whether differences exist in the subscales of the I.ROC for the CRT sample that suggest relative strengths of the service and areas for improvement.

## Methods

This evaluation used a retrospective cohort design using quantitative data from a CRT’s electronic records of I.ROC scores. Consistent with HRA guidance (The Health Research Authority 2017), ethical approval for this evaluation was not required; however, the evaluation was registered with the local NHS trust on their Quality Management System after being reviewed and approved by the Clinical Research Manager.

### Participants

Adults, aged 18 years and older, supported by the CRT from its inception in April 2020 until the discharge of the first referral in November 2021 were included in the analyses. Initial I.ROC questionnaires were completed once the referral was accepted and the clinician had developed rapport with the person. Thereafter, I.ROC questionnaires were completed quarterly.

Referrals to the CRT are individually reviewed in Multi-Disciplinary Team (MDT) clinical review meetings held weekly. CRT differs from Community Mental Health Teams (CMHTs) in that CRT reviews referrals from people currently being supported in inpatient services who are due to transition into the community and who have been identified as needing support over and above what is expected from CMHTs. Referrals that exceed the severity threshold for Improving Access to Psychological Therapies (IAPT) but do not meet the threshold for other secondary care services such as eating disorders or personality and complex trauma are eligible for support from CRT.

### Measure

The I.ROC questionnaire is a process measure of mental health recovery that has excellent reliability as measured by the Interclass Correlation Coefficient, ICC = 0.909, and good concurrent validity; significantly correlated with known measures of recovery such as the Recovery Scale and the Behaviour and Symptom Identification Scale (Dickens et al. 2019; Monger et al. 2013). The I.ROC consists of four subscales creating the HOPE acronym: Home, Opportunity, People, and Empowerment. Each subscale has three questions scored on a six-point Likert scale from one (never) to six (all the time), resulting in a minimum score of 12 and maximum of 72. Higher scores on the I.ROC indicate better wellbeing, and improvements across subsequent I.ROC questionnaires suggest recovery. In the Home subscale, for example, one of the items, life skills, asks the person how often, in the last three months, they have felt they have the skills they need to look after themselves. Within this item, the staff member will reflect with the person how they manage cooking, cleaning, money skills, bills, shopping, personal care, and being a good neighbour. The I.ROC provides opportunity for the inclusion of qualitative information to support the Likert rating. Such that barriers or issues that arise that impact on the score can be resolved and subsequent I.ROC reviews can reflect the progress made. For example, if a person requests support in managing their finances, a staff member can work with them to improve their budgeting skills. The subsequent I.ROC, three months later, should then reflect the increase in life skills given the support provided in helping the person manage their finances through budgeting skills. The person therefore, learns skills that enable them to become more confident and live successfully in the community. The other two items in the Home subscale ask about mental health and safety and comfort. In the Opportunities subscale the three items ask how the person feels about their physical health, exercise and activity, and purpose and direction. In the People subscale the three items ask about the person’s personal network, social network, and how they value themselves. Finally, in the Empowerment subscale, this asks the person how they have felt about their participation and control, self-management, and their hope for the future.

### Analyses

To fulfil objective one, I.ROC data available in the literature for clinical and non-clinical populations was necessary in order to calculate the RCI and CSC for individuals in the CRT sample. I.ROC data reporting on a clinical population comprised of 171 community-dwelling adults in Scotland accessing support from mental health teams provided suitable data (Rudd 2018). Of the 171 people, 92 (54%) were men, the age range was from 15 to 79 years with a mean age of 46 years. No data on ethnicity was reported. Rudd (2018)’s non-clinical population comprised of 70 students and staff at a Scottish University, as well as 104 staff working in a mental health service, resulting in a total sample of 174 non-clinical participants. Of the 174 people, 135 (78%) were women, the sample age range was from 18 to 80 years with a mean age of 35 years. No data on ethnicity was reported. The psychometric data available from Rudd (2018) was inputted into an excel spreadsheet designed to calculate the RCI and CSC (Morley and Dowzer 2014) alongside the individual I.ROC data available from the CRT. Table 1 presents the psychometric properties of I.ROC data in clinical and non-clinical samples (Rudd 2018).

**Table 1:** Psychometric properties of clinical and non-clinical samples from Rudd (2018) needed to calculate RCI and CSC

I.ROC	n	Mean	SD	Minimum	Maximum	Cronbach’s alpha
Clinical	171	44.9	10.8	18	72	0.86
Non-clinical	174	54.5	10.8	22	72	0.92

Prior to calculating the RCI and CSC for individuals in the CRT sample, it was necessary to ensure the sample drawn from the literature and the CRT sample were comparable and did not significantly differ. Individual I.ROC total scores for the CRT sample were exported into IBM Statistical Package for the Social Sciences (SPSS; IBM 2020) and checked for any missing data. Following this a one sample t-test was conducted to determine if any significant differences existed between the CRT sample and the published clinical sample. If no significant difference was found between the two samples, then RCI and CSC analyses could be completed and any subsequent changes could not be attributed to pre-existing differences.

To fulfil objective two, individual I.ROC subscale scores for the CRT sample were exported into SPSS. A paired samples t-test was conducted to determine if any differences were evident between I.ROC subscales between review periods. Differences observed here would suggest the CRT were performing better in some subscales compared to others, and lead to recommendations of where improvements could be made for the service.

The authors declare no known conflicts of interest and certify responsibility for this evaluation.

## Results

A total of 31 people supported by the CRT had two completed I.ROC questionnaires between April 2020 and November 2021. Of these 31 people, 14 people had three completed I.ROC questionnaires. There was no missing data for any of the items on completed I.ROC questionnaires. Of the 31 people, 26 (84%) were men, the age range was from 21 to 66 years, with a mean age of 42 years. Most, n = 30 (97%), people identified as White British, with one person identifying as Any Other White Background.

### Objective 1

A one sample t-test was conducted to determine whether or not the CRT sample and clinical sample from Rudd (2018) were significantly different. The CRT sample was normally distributed as evidenced by non-significant results from tests of normality Kolmogorov–Smirnov, D (31) = 0.078, p = 0.20, and Shapiro–Wilk, W(31) = 0.969, *p* = 0.497.

The one-sample t-test using data from the 31 completed I.ROC questionnaires (M = 44.58 SD = 9.16) and the published mean of the sample (M = 44.9) from Rudd (2018) showed no statistically significant difference between the two samples [t(30) = −0.194, *p* = 0.847].

#### First Review Period: First I.ROC to Second I.ROC

Descriptive statistics report the first I.ROC questionnaire mean was 44.58 (SD = 9.16), while the second I.ROC questionnaire mean increased to 54.58 (SD = 7.92). The RCI and CSC were calculated and the results are presented in Table 2. In this sample, 17 people showed a reliable positive change in their I.ROC questionnaire scores. Three people showed clinically meaningful improvement indicating their scores had moved to within a non-clinical population distribution rather than a clinical population.

**Table 2:** Reliable Change Index and Clinically Significant Change for each person’s I.ROC score

Participant	First I.ROC total score (n = 31)	Second I.ROC total score (n = 31)	Third I.ROC total score (n = 14)
1	43	53 ^R+^	–
2	53	59	69^R+^
3	56	60	34^R−^
4	52	66 ^R+^	70
5	51	49	51
6	37	43	–
7	41	54^R+^	54
8	51	50	53
9	46	50	–
10	31	62^R+^	–
11	47	60^R+^	–
12	54	54	–
13	58	61	–
14	30	58^R+^	–
15	43	51^R+^	–
16	47	65 ^R+^	–
17	43	53^R+^	57
18	69	69	–
19	47	52	–
20	33	38	40
21	39	66^R+^	59^R−^
22	48	56^R+^	55
23	32	43^R+ C+^	–
24	35	40	–
25	48	47	45
26	38	58^R+^	–
27	35	47^R+C+^	52
28	46	63^R+^	68
29	40	49^R+C+^	55
30	55	60	–
31	34	56^R+^	–
RCI +	–	17	1
CSC +	–	3	0
RCI −	–	0	2

R^+^ indicates a significant RCI towards improvement. C^+^ indicates a significant CSC towards recovery. R^−^ indicates a significant RCI towards deterioration

#### Second Review Period: Second I.ROC to Third I.ROC

Descriptive statistics for the 14 people who had three completed I.ROC questionnaires are reported. The second I.ROC questionnaire mean was 54.07 (SD = 8.11) while the third I.ROC questionnaire mean increased slightly to 54.43 (SD = 10.38). In this sample, one person showed a reliable positive change indicating their I.ROC questionnaire score improved. Two people showed a reliable negative change indicating their scores decreased, 11 people showed no change, and no-one showed clinically meaningful improvement.

### Objective 2

It was thought that differences between the I.ROC subscales across the two review periods would indicate relative strengths of CRT in supporting people in some areas than others on their recovery journey. To assess for this, a paired sample t-test was conducted for each subscale between the first review period, i.e., from the first I.ROC to the second I.ROC (see Table 3). During this first review period, all subscales indicated a significant difference in the direction of improvement i.e., all subscale scores increased. This suggests no relative strengths or areas for improvement as the CRT were able to achieve progress across the four subscales.

**Table 3:** Paired samples t-test for I.ROC subscales across the first and second I.ROC reviews

	Mean (standard deviation)	t	*P*
Home^1^	11.52 (2.74)		
Home^2^	14.26 (2.32)	−5.467	***P* < .001**
Opportunity^1^	11.16 (3.11)		
Opportunity^2^	12.97 (2.24)	−.633	***P* = *.*004**
People^1^	10.35 (3.18)		
People^2^	12.77 (2.92)	−1.379	***P* < .001**
Empowerment^1^	11.55 (2.74)		
Empowerment^2^	14.58 (2.32)	−1.930	***P* < .001**

^1^indicates subscale data at first I.ROC

^2^indicates subscale data at second I.ROC. Bold values indicate *P* < .05

This was followed by a paired samples t-test for each subscale between the second review period, i.e., from the second I.ROC and the third I.ROC (see Table 4). During this review period, no statistically significant differences were observed between the subscales; the Home and People subscale scores increased, Opportunity remained unchanged, and Empowerment decreased. This suggests that the CRT were able to maintain the progress made from the previous review period.

**Table 4:** Paired samples t-test for I.ROC subscales across the second and third I.ROC reviews

	Mean (SD)	t	*P*
Home^1^	14.50 (2.21)		
Home^2^	14.64 (2.41)	−.314	.759
Opportunity^1^	12.79 (2.12)		
Opportunity^2^	12.79 (3.31)	.000	1.000
People^1^	12.57 (2.71)		
People^2^	12.93 (2.59)	−.528	.606
Empowerment^1^	14.21 (2.64)		
Empowerment^2^	14.07 (3.29)	.193	.850

^1^indicates subscale data at second I.ROC

^2^indicates subscale data at third I.ROC

## Discussion

This service evaluation aimed to assess whether a person supported by a CRT made progress towards mental health recovery as evidenced by improvements in their I.ROC scores, where I.ROC is a measure of mental health recovery. As outlined previously, there is no unifying definition of recovery in mental health nor is there one model of recovery service provision (Killaspy et al. 2005). To investigate this aim we developed two objectives. The first objective was to calculate individual reliable and clinical change indices to show if recovery was made as indicated using the I.ROC. The second objective was to detect any difference between the I.ROC subscales that would indicate areas the CRT service does well and areas for improvement.

Although there were some differences between the samples from the CRT and Rudd (2018): the CRT sample had a higher proportion of men than Rudd (2018), 84% and 54% respectively, and the CRT sample had a narrower age range than the sample from Rudd (2018), 21–66 years and 15–79 years, respectively. Where the older starting age range likely reflects the fact that adult inpatient services in England are commissioned to work with adults from 18 years upwards (Penfold et al. 2019). These differences were not statistically significant and therefore the samples were appropriate to use in the analysis to calculate the RCI and CSC indices.

Objective 1: During the first review period, 17 out of 31 people (55%) showed a reliable improvement, and three people (9.7%) showed clinically significant change suggestive of recovery. Comparing the CRT results to a study looking at the clinically meaningful change of 1,830 patients following an inpatient stay, 12.8% people showed reliable improvement (positive RCI), 41.8% of people recovered (positive RCI and CSC), 43.6% had no change, and 1.7% declined (Newnham et al. 2007). Of the four outcome measures: Medical Outcomes Short Form Questionnaire (SF-36); Depression Anxiety Stress Scale (DASS-21); Quality of Life Enjoyment and Satisfaction Questionnaire (Q-LES-Q); and the Health of the Nation Outcome Scale (HoNOS), none measure mental health recovery and none appear on the systematic review of recovery measures conducted by Penas and colleagues (2019). Although the study by Newnham and colleagues (2007) had a higher proportion of people showing clinically significant change than the CRT sample, the study was conducted in an inpatient sample, therefore two considerations are worth highlighting. Firstly, people are usually admitted to an inpatient unit when they are at their most distressed and therefore there is a large scope for their mental health to improve. Secondly, patients at the point of discharge would be expected to have shown signs of improvement. In another study, this time of 593 community-dwelling adults accessing outpatient support for their mental health, 23.6% showed improvement (a positive RCI) and 18% showed a decline (a negative RCI; Eisen et al. 2007). Here the authors used the Behaviour and Symptom Identification Scale (BASIS-24), two self-report measures of change: mental health and retrospective transition, as well as the Global Assessment of Function. None of these measures were developed to measure recovery although the authors aimed to measure change following mental health treatment, their focus was inexplicably on clinical symptoms, for example, psychotic symptoms, self-harm, etc. (Eisen et al. 2007). The CRT results therefore compare favourably to published results of both inpatient and outpatient settings. From the second to the third I.ROC questionnaire the results were mixed. Although, there was not a consistent increase in reliable and clinical change, the progress achieved was maintained i.e., there was not a large decline in I.ROC scores (only two people showing a reliable decline in their scores with one person showing a reliable improvement).

Objective 1 used reliable and clinically meaningful indices to determine if people made progress in their recovery journey. The results suggest that the CRT were successful in supporting people to adapt to community living and meet their basic needs such as housing, income, sourcing meaningful activities, and creating social networks in the community. This priority on basic needs is consistent with Maslow’s hierarchy of needs concept where subsequent needs can only be met when more basic needs are met (Maslow 1943). For example, physiological needs must be met before higher, such as psychological needs like self-esteem can be prioritised. This concept of a hierarchy of needs has been taken forward in designing recovery-oriented services where greater emphasis is placed on care coordinating to meet basic needs first before supporting a person to develop connectedness in their community, positive relationships, and pursuing meaningful employment and activities (Isaacs et al. 2019). The service implications of this evaluation indicate a holistic and individualised approach to recovery is necessary for progress. This is consistent with the concept of a hierarchy of needs put forward by Maslow (1943) as well as considering the contributors of social exclusion that need to be addressed for recovery to take place (Karadzhov 2021).

Objective 2: Data for the four subscales, Home, Opportunity, People, and Empowerment in the two review periods were analysed for any differences that may indicate relative strengths or areas for improvement for the CRT. Consistent with the results from objective 1, all subscales showed significant improvement in the first review period. Whereas, all subscales showed no significant difference in the second review period, largely consistent with the results of objective 1 where there was limited change in individual reliable and clinically meaningful change indices in the second review period.

At a service level, the results demonstrate that the CRT maintained the progress made for the people they support. Consideration for the wider context is necessary not to lose perspective of the overall recovery journey people experience. The people supported by the CRT had all received treatment at an inpatient service and were deemed well enough to be discharged. They then successfully transitioned into a community setting; a significant step in the recovery journey, particularly given the risks associated with increased mortality in the first three months (Musgrove et al. 2022). Therefore, the level of recovery that can be expected is likely to be of a modest nature and highly individual.

The I.ROC measure has been shown to be useful to evidence recovery at an individual and service level, arguably core qualities needed in a routine outcome measure (Happell 2008). At an individual level, people supported by CRT have a say in what matters to them and they have the flexibility to reflect on their recovery journey using the subscales within the I.ROC. Although, they are supported by staff to complete the I.ROC quarterly, they have ownership of their I.ROC. At a service level, the I.ROC allows for a dynamic assessment of progress. Where progress plateaus, the subscales allow for a granulated investigation into where more resources might provide the greatest impact. Where progress is achieved, there is confidence to say that the CRT are providing a holistic and person-centred approach to mental health recovery. This is consistent with the findings that the use of the I.ROC in survivors of psychological trauma was personally meaningful as well as being useful to the service to measure progress (Rudd et al. 2020).

### Recommendations

This service evaluation has shown the CRT to successfully support people in their mental health recovery. Although, this progress was largely maintained at the second review period, limited subsequent progress was evident. It is possible that people may need more intensive support when initially moving from the highly structured environment of a mental health hospital to the community. However, after this initial period of adaption, it might be that a continued high-level involvement of the CRT may reinforce the perception that the person is unable to function in the community, leading to a stagnation of I.ROC scores. Conversely, it might be that after the initial adaption to community life, the person feels overwhelmed living in the community and requires additional support from the CRT. A recommendation here is to determine what people need at the point of the second I.ROC review i.e., at the six-month point that would enable them to continue making progress in their mental health recovery. Given that mental health recovery is deeply personal, future research might use qualitative methodologies as these are most likely to be the most appropriate way to identify themes and shed light on what it is people need most to continue to make progress towards recovery (Peters 2010).

Due to the retrospective nature of this service evaluation, the depth and breadth of questions we were able to ask of the available data were limited. Moving forward, a recommendation would be for services to set out evaluation aims a priori, thus allowing for theoretically driven knowledge to be generated rather than relying on empirical observations. For example, a future service evaluation could systematically capture the experience of using the I.ROC from the perspective of the person, their family, and/or carers. This would inform whether the I.ROC alone is sufficient to measure mental health recovery or whether additional measures are needed to fully capture the recovery journey from the different stakeholders. Practically, this should not be too burdensome for the person being supported or their family or carers and can be incorporated as a feedback survey at regular points or at the point of discharge where the person, or those in their system, can reflect back on the journey about what was helpful and what would have been helpful.

### Limitations

A limitation of this evaluation was the use of retrospective data which limited the questions we could ask of the data. Fortunately, in this evaluation there was no missing data, however collating the necessary information in order to conduct analysis could be prospectively initiated to facilitate timely data analysis and remove time barriers. As this was a service evaluation using existing data, the sample size was limited to what was available at the time of analysis. In future, this evaluation should be repeated with a larger sample to assess the CRT’s progress. It would also be useful to include analysis of subsequent I.ROC reviews i.e., more than two I.ROC review points, to track the CRT’s progress longitudinally. Given this CRT is fairly new, less than five years in operation, this service evaluation provides a baseline from which subsequent evaluations can build on.

Although the I.ROC is recommended to be completed quarterly, this service evaluation did not explicitly assess whether I.ROC reviews were being conducted consistently. Given the disruption of the COVID-19 pandemic and frequent changes in service policy regarding infection control in response to government lockdowns and restrictions, it would seem likely that I.ROC reviews were completed on an ad hoc basis. This has consequences when trying to compare individuals’ progress where some people may have had more time during an I.ROC review period to make progress due to a delay in staff being able to conduct the review.

The cohort under investigation in this service evaluation was limited particularly in terms of ethnic, cultural, and racial diversity. All people included in this evaluation identified as either White British or any other White. It is therefore not known if the recovery journey of people from different ethnic, cultural, or racial backgrounds is similar or different. It is of concern that groups of people may not be accessing mental health services which they may benefit from. This is an ongoing issue in mental health service provision where, despite being aware of equality issues, commissioners do not take this into account when commissioning services (Murray 2020).
